# Interaction of brain endothelial cells with T cells: implications for progression and therapeutic strategies of multiple sclerosis

**DOI:** 10.3389/fneur.2026.1721076

**Published:** 2026-02-19

**Authors:** Yan Zhao, Dongqi Zhu

**Affiliations:** 1 Institute of Clinical Research and Translation, Jinan Third People’s Hospital, Jining Medical University, Jinan, China; 2 Department of Pharmacy, Shandong Cancer Hospital and Institute, Shandong First Medical University and Shandong Academy of Medical Sciences, Jinan, China

**Keywords:** brain endothelial cells, multiple sclerosis, progression, T cells, therapeutic strategies

## Abstract

The dynamic interplay between brain endothelial cells (BECs) and T cells is a key event in the pathogenesis of multiple sclerosis (MS). This process allows the extravasation of T cells from the peripheral circulation into the central nervous system (CNS), thereby triggering neuroinflammation and tissue damage. In MS, activated BECs facilitate T cell activation, recruitment, and CNS infiltration by upregulating major histocompatibility complex (MHC) molecules, cell adhesion molecules (CAMs), and chemokines. In response, T cells interact with BECs by expressing corresponding ligands, thereby modulating the immunoregulatory and barrier functions of BECs. This cross-talk between BECs and T cells significantly increases the complexity of MS treatment. Therefore, this review systematically summarizes the mutually reinforcing interactions between BECs and T cells based on existing research and highlights recent therapeutic strategies for either BECs or T cells that show promise in achieving favorable outcomes for treating MS.

## 1 Introduction

Multiple sclerosis (MS) is a chronic autoimmune disease characterized by T cell infiltration, demyelination and axonal damage. Recent studies have found that blood–brain barrier (BBB) destruction is an early feature of MS pathologies, which predates the entry of pathogenic T cells into the central nervous system (CNS) (1). Therefore, BBB disruption is considered a key driver in the progression of MS. Clinically, gadolinium-enhanced MRI is used to assess BBB dysfunction in CNS and the outcomes serve as diagnostic criteria for MS (2, 3). Histopathological evidence also confirms BBB abnormalities in MS lesions (4, 5), further highlighting the critical role of the BBB in the progression of MS.

BBB is composed of a monolayer of brain endothelial cells (BECs) along with adjacent perivascular cells, including astrocytes and pericytes (6). This structure protects the CNS from harmful blood-borne, exogenous, and endogenous substances, thereby maintaining the homeostasis of the neural microenvironment (7). Generally, BECs, as the core component of the BBB, play an important role in maintaining its barrier properties. It is noteworthy that BECs also possess non-professional antigen-presenting and immunomodulatory functions (8). In humans and most mammals, BECs constitutively express MHC molecules but typically lack the expression of the co-stimulatory molecules CD80 and CD86. However, *in vitro* studies suggest that under specific inflammatory stimulation, BECs are activated and their immunomodulatory functions are significantly enhanced. On one hand, BECs upregulate the expression of immunomodulatory molecules (9, 10). On the other hand, adhesion molecules like ICAM-1 and VCAM-1, whose expression is significantly upregulated, not only mediate firm T cell adhesion but also provide crucial co-stimulation alongside MHC–peptide recognition (11). These molecules collectively form a complex network of signal interactions. By engaging with their respective ligands on T cells, they participate in the recruitment, differentiation, and proliferation of T cells, thereby playing a central role in the initiation and amplification of neuroinflammation.

Recent studies have confirmed that T cells and their subsets play a critical role in the pathogenesis of MS. During MS development, the number of T cell infiltration in CNS is closely correlated with tissue injury of patients (12). It is important to note that the entry of T cells into the central nervous system parenchyma during neuroinflammation is a multi-step process. Following initial capture and adhesion, T cells first enter and are retained in the perivascular space. This compartment is filled with cerebrospinal fluid and contains professional antigen-presenting cells such as perivascular macrophages, constituting a critical immune checkpoint. Subsequently, T cells must cross the glial limitans, formed by astrocytic endfeet, to finally infiltrate the neural tissue (13). Within this infiltration cascade, the interaction between pathogenic T cell subsets and BECs serves as a key driver of the pathological process. Among these, Th1, Th17, and CD8⁺ T cells have been demonstrated to interact with BECs in specific ways. Th1 cells strongly activate pro-inflammatory signaling pathways in BECs through the production of interferon-gamma (IFN-*γ*), disrupting tight junctions. Th17 cells induce the expression of chemokines via secretion of interleukin-17 (IL-17) and interleukin-22 (IL-22), thereby recruiting other immune cells and exacerbating neuroinflammation. Additionally, CD8^+^T cells can directly recognize antigens presented by BECs and mediate cytotoxic effects. Activated T cells boost the destruction of BBB upon adhesion and during or after trans-endothelial migration, leading to the influx of peripheral immune cells into the CNS (14). Therefore, the specific dialog between BECs and T cells constitutes a central mechanism driving neuroinflammation and disease progression.

This review is intended to provide a comprehensive summary of the interactions between BECs and T cells based on current research. We further discuss the therapeutic strategies for targeting BECs or T cells and highlight the potential benefits of attenuating the interplay between the two cell types. Our goal is to provide a powerful reference for the future development of clinical therapies targeting BECs or T cells.

## 2 Characteristics of BECs

BECs play crucial roles in maintaining BBB integrity by intercellular junction complexes, including tight junctions (TJs), adherens junctions (AJs) and gap junctions (GJs). TJs are composed of transmembrane proteins (occludin, claudins), zonula occludens (ZO) and junction adhesion molecules (JAMs), which interact with actin cytoskeleton to seal the paracellular space. Similarly, AJs, composed of transmembrane proteins (VE-cadherin) and cytoplasmic proteins (*β*-catenin), are linked to actin cytoskeleton to control the endothelial barrier permeability. Among them, VE-cadherin mediates intercellular adhesion, while *β*-catenin provides structural support and regulates junctional stability (15). In addition, GJs, formed by hexamers of connexin (Cx) subunits, mediate intercellular communication and modulate BEC function. Under inflammatory conditions, abnormal expression of Cx is closely associated with BEC dysfunction (16). Notably, impaired BEC function can increase BBB permeability, which allows the extravasation of peripheral immune cells into the brain tissue. Hence, protecting the BEC barrier function is essential for reducing BBB permeability and maintaining the homeostasis of CNS.

BECs are not only important gatekeepers but also have immunomodulatory properties containing immune cell recruitment and antigen-present. BECs express adhesion molecules and chemokines to facilitate immune cell recruitment and extravasation into sites of injury (17). Under pathological conditions, proinflammatory mediators (including IL-1β and TNF-*α*) induce the upregulation of ICAM-1 and VCAM-1 on BECs, which are essential for immune cell adhesion and subsequent infiltration into the brain parenchyma (18). Meanwhile, chemokines also play key roles in immune cell recruitment. Activated BECs has been shown to secret chemokines such as CCL2, CCL19, CXCL10, which attract T cells to invade the CNS via paracellular routes. In addition to recruiting immune cells, BECs act as non-professional antigen-presenting cells that can induce T cell responses by expressing MHC and costimulatory molecules. Studies have reported that BECs can ingest and present myelin antigens to T cells via MHC molecules (19). The costimulatory molecules on BECs bind to corresponding receptors on T cells, supplying the essential second signal for activation and differentiation. These activated T cells release inflammatory cytokines, intensifying neuroinflammation and exacerbating demyelination.

In summary, BECs occupy a crucial role in the pathogenesis of MS through their dual functions: maintaining structural integrity of the barrier and actively regulating immune responses. The integrity of their intercellular junctional systems forms the structural basis for CNS homeostasis, while their capabilities in antigen presentation and immune cell recruitment directly initiate and amplify neuroimmune responses. Thus, exploring the immune regulatory mechanisms of BECs is significant for understanding the immune pathogenesis of MS and identifying new therapeutic targets.

## 3 The regulatory roles of BECs on T cells

**Figure 1:**
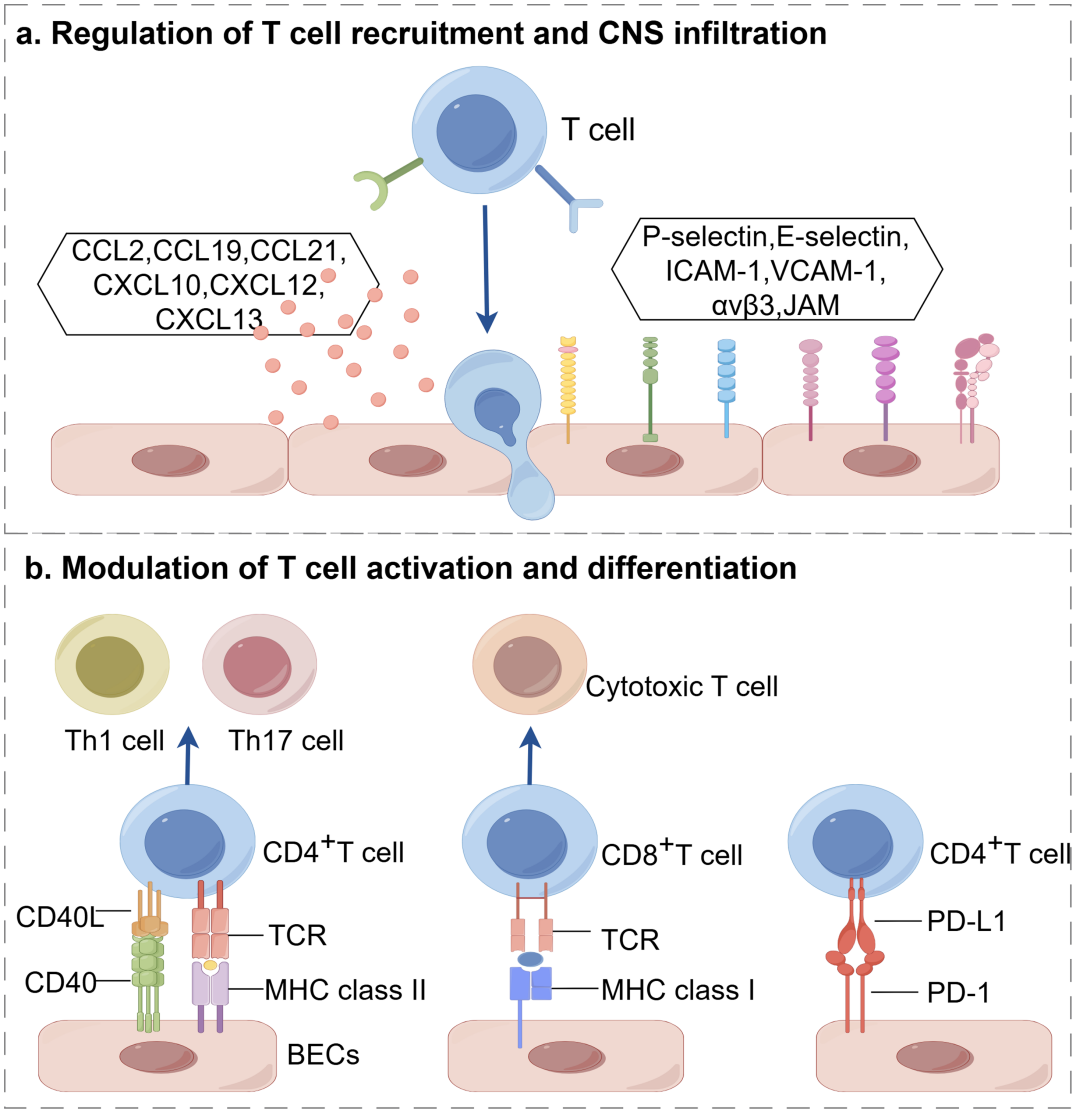
Activated BECs dynamically regulate T cells in multiple sclerosis, including **(a)** recruitment and CNS infiltration, and **(b)** activation and differentiation. (created with Home for Researchers, https://www.home-for-researchers.com/).

The impact of CNS infiltration and functional changes in T cells on MS progression has been repeatedly emphasized. In recent years, the regulation of T cells by BECs has attracted increasing attention (Table 1; Figure 1). As integral components of BBB, BECs also function as non-professional antigen-presenting cells (APCs). They actively recruit circulating T cells, enhance their migration across the BBB into the CNS, and further promote the activation and differentiation of T cells, thereby intensifying the damage to the myelin sheath.

**Table 1:** The regulatory effect and mechanism of BECs on T cells.

Effect	BEC-associated molecules	T cell-associated molecules	Ref.
Recruitment	CCL2 CCL19, CCL21 CXCL10 CXCL12 CXCL13	CCR2 CCR7, CXCR3 CXCR3 CXCR4, CXCR7 CXCR5	(22) (23, 24) (27, 28) (31, 32, 34) (35)
CNS infiltration	P-selectin, E-selectin ICAM-1, VCAM-1 integrin α3 MCAM JAM-B DICAM	PSGL-1, ESL-1 LFA-1, VLA-4 laminin α5 ST14 VLA-4 integrin αvβ3	(37) (40, 41) (42) (43) (45) (38)
Activation and differentiation	MHC CD40, ICOSL PD-1	TCR CD40L, ICOS PD-L1	(19, 49, 50) (48) (51)

### 3.1 Regulatory dynamics of BECs on T cell recruitment and CNS infiltration

Chemokines are a set of conserved small proteins, 8–10 kDa, that are critical mediators of immune cell recruitment and migration. In MS, chemokines secreted by BECs bind to specific receptors on T cells, directly regulating their trafficking and CNS infiltration. Prior studies have identified several key players in this process, including CCL2, CCL19, CCL21, CXCL10, CXCL12, and CXCL13. For instance, in MS patients or animal model experimental autoimmune encephalomyelitis (EAE), the expression of p-glycoprotein (P-gp), an ATP-binding cassette transporter, is reduced in BECs (20, 21). Kooij et al. found that the trafficking of cytotoxic CD8^+^T cells is dependent on BEC- derived P-gp, which upregulates the secretion of CCL2 during EAE. Specific knockout of CCL2 in BECs can significantly diminish CD8^+^T cell accumulation (22). BECs also secret CCL19 and CCL21 to recruit T cells that express CCR7 and CXCR3, which are implicated in initiating and sustaining CNS inflammation in EAE model. These chemokines can further upregulate lymphocyte function-associated antigen-1 (LFA-1) and very late antigen-4 (VLA-4) by activating G-protein-coupled receptor, thereby promoting T cell adhesion to BECs (23). Blocking CCL19 and CCL21 can effectively decrease the adherence of T cells to BECs in EAE model (24). In MS patients, elevated levels of CXCL10 expression closely correlate with disease activity (25). Wang et al. found that IFN-*γ* activates the STAT1 pathway in BECs, thereby promoting CXCL10 secretion in a Rac1-dependent manner (26). CXCL10 then binds to CXCR3 on activated T cells to facilitate their chemotaxis toward the sites of inflammation (27, 28). Under physiological conditions, the chemokine CXCL12 exhibits polarized distribution at the BBB, primarily localized to the abluminal side of BECs (29). This spatial expression pattern helps maintain immune isolation of CNS by preventing aberrant leukocyte infiltration. However, under pathological conditions, this polarized distribution of CXCL12 is disrupted, ultimately promoting the invasion of autoreactive immune cells and driving neuroinflammation (30). McCandless et al. found that CXCL12 is internalized and redistributed to the luminal side of endothelial cells in active MS lesions. This spatial relocalization establishes a chemotactic gradient directed toward the vascular lumen, significantly enhancing the infiltration capacity of CXCR4^+^T cells into the central nervous system and thereby exacerbating tissue damage such as demyelination (31, 32). These findings have been consistently validated in EAE model, collectively demonstrating that the polarity switch of CXCL12 during autoimmunity is a key mechanism mediating leukocyte capture and infiltration at the luminal surface of microvessels (33). Further research has revealed the regulatory role of CXCR7 in this process. CXCR7, functioning as an alternative receptor for CXCL12, primarily sequesters and degrades CXCL12 through endocytosis, thereby finely regulating its bioavailability in the local microenvironment. In EAE model, endothelial expression of CXCR7 is significantly upregulated, and antagonizing CXCR7 not only blocks the endothelial internalization and lysosomal degradation of CXCL12 but also markedly alleviates disease severity by reducing CD4^+^T cell infiltration. The mechanism underlying this protective effect, beyond correcting the disrupted spatial distribution of CXCL12, may also be partially attributed to the downstream downregulation of VCAM-1 (34). CXCL13, regarded as a biomarker of MS severity, exacerbates neuroinflammation by binding to its specific receptor CXCR5 (35, 36). Together, these chemokines form an intricate network that regulates the recruitment and CNS infiltration of T cells in MS.

The CNS infiltration of T cells is a sequential and multistep process that involves capture, rolling, firm adhesion, crawling and diapedesis. This cascade is mediated by selectins, integrins, and immunoglobulin superfamily members expressed on BECs. P-selectin and E-selectin on activated BECs promote the initial capture and rolling of T cells through binding to P-selectin glycoprotein ligand-1 (PSGL-1) and E-selectin ligand-1 (ESL-1) (37). Abnormal integrin expression on BECs also positively correlates with immune cell infiltration in MS. A recent study discovered that the dual immunoglobulin domain-containing cell adhesion molecule (DICAM), preferentially expressed on Th17 cells, facilitates Th17 cell trafficking across the BBB via interaction with its ligand αvβ3 (38). Members of the immunoglobulin superfamily, including ICAM-1, VCAM-1, and melanoma cell adhesion molecule (MCAM), are upregulated in BECs during MS/EAE and promote leukocyte adhesion and transendothelial migration (39). ICAM-1 and VCAM-1 are responsible to stabilize the adhesion and arrest of T cells via binding to LFA-1 and VLA-4, respectively (40, 41). Furthermore, integrin interactions with laminin initiate the cell migration process. Park et al. confirmed that integrin α3, selectively expressed on Th17 cells in EAE, boosts Th17 cell arrest on vascular endothelium through binding to laminin5 on BECs (42). Another study found that MCAM is preferentially expressed on BECs in MS patients and EAE model. *In vitro and in vivo* experiments have demonstrated that MCAM binding to ST14 on Th1 and Th17 cells promotes their cellular trafficking and EAE development (43). JAMs, components of tight junctions, also modulate vascular permeability and leukocyte transmigration. The absence of JAM-A immunostaining in lesion areas of MS patients, coupled with observed BBB leakage, suggests a critical role for JAM-A in BBB integrity (44). During the multi-step process of T cell extravasation, VLA4 plays a dominant role. Studies have suggested that the mechanism by which CD8^+^T cells utilize this integrin to interact with the BBB differs from that of CD4^+^T cells and may involve the tight junction protein JAM-B. In this regard, the work of Pareja et al. provides a more precise explanation: their experiments demonstrated that while the absence of JAM-B did not alter the adhesion of CD8^+^T cells to the BBB or their ability to complete transendothelial migration, it significantly impaired their crawling speed on the luminal surface of blood vessels (45). This finding indicates that JAM-B may facilitate the passage of CD8^+^T cells across the BBB by enhancing their motility.

In summary, BECs precisely control the recruitment and CNS infiltration of T cells through a coordinated program involving chemokine gradients and adhesion molecule cascades. The specific combination of chemokines released determines which T cell subsets are recruited, while the spatiotemporal expression of adhesion molecules guides their multi-step migration across the BBB. Disruption of this sophisticated regulatory system is a hallmark of MS neuroinflammation.

### 3.2 Modulation of BECs on T cell activation and differentiation

The antigen-dependent activation of T cells requires two synergistic signals. The first signal is antigen-specific, delivered through the engagement of the T cell receptor (TCR) with MHC-antigen peptide complexes presented by APCs. The second signal is provided by costimulatory molecules on APCs (such as CD80, CD86) binding to receptors such as CD28 on T cells. BECs, acting as non-professional antigen-presenting cells, deliver stimulatory signals to T cells by processing and presenting antigens (46, 47). It has been demonstrated that TNF-*α* induces the upregulation of MHC class II molecules and CD40 on BECs, enabling them to present myelin antigens to CD4^+^T cells and promote the transendothelial migration of antigen-specific Th1 and Th17 cells (19). Wheway et al. revealed that BECs can internalize soluble antigens via macropinocytosis *in vitro* and present them via high levels of MHC class II molecules in conjunction with costimulatory molecules (CD40 and ICOSL), thereby amplifying the proliferation of CD4^+^T cells (48). Furthermore, BECs-expressed MHC class I molecules play a pivotal role in the interaction with CD8^+^T cells at the BBB. Their primary function is to mediate the antigen-specific arrest of circulating effector CD8^+^T cells on the luminal surface. This interaction is crucial for facilitating their subsequent CNS infiltration, as blocking MHC class I effectively reduces effector CD8^+^T cell entry into the brain (49). Aydin et al. provide a mechanistic detail to this process. They demonstrated that antigen presentation by BECs via MHC class I not only arrests effector CD8^+^T cells but also abolishes their subsequent crawling and diapedesis. This prolonged arrest and engagement ultimately lead to BECs apoptosis and focal BBB disruption. Notably, they also found that inflamed BECs can support the activation and proliferation of naïve CD8^+^T cells in an MHC class I-dependent manner, similarly culminating in endothelial damage (50). In addition to providing costimulatory signals, BECs also express coinhibitory molecules to finely regulate T cell responses. PD-1 is a key immune checkpoint molecule that, upon binding to its ligand PD-L1, transmits inhibitory signals to suppress the activation and proliferation of T cells. To elucidate the specific role of this pathway within the neuroimmune microenvironment, Klota et al. systematically investigated the function of PD-1/PD-L1 signaling in BEC-T cell interactions and lesion distribution using the opticospinal EAE (OSE) model. By specifically ablating PD-L1 on T cells while preserving its expression on antigen-presenting cells, the researchers observed a significant enhancement in the ability of T cells to induce BEC dysfunction in an *in vitro* BBB model, an effect strictly dependent on direct cell–cell contact. Mechanistic studies further revealed that PD-L1-deficient CD4^+^T cells markedly promoted BEC apoptosis via increased secretion of IFN-*γ* and granzyme B, ultimately leading to sustained elevation of BBB permeability both *in vitro* and *in vivo* (51). This work not only confirms that the activation state of T cells is determined by a dynamic balance between costimulatory and coinhibitory signals, but also demonstrates that intervention targeting even a single immunoregulatory molecule can alter the capacity of T cells to induce focal BBB dysfunction, thereby providing a novel theoretical framework for understanding the spatial distribution characteristics of MS lesions.

In summary, BECs function as pivotal gatekeepers of T cell immunity within the neurovascular unit, shaping T cell activation and differentiation. By providing MHC-mediated antigen presentation, costimulatory signals, and immune checkpoint modulation, BECs establish a dynamic link between innate barrier function and adaptive immune responses, profoundly influencing the delicate balance between neuroprotective immunity and pathological autoimmunity in the progression of MS.

## 4 Influence of T cells on BECs function of barrier and immunomodulation

**Figure 2:**
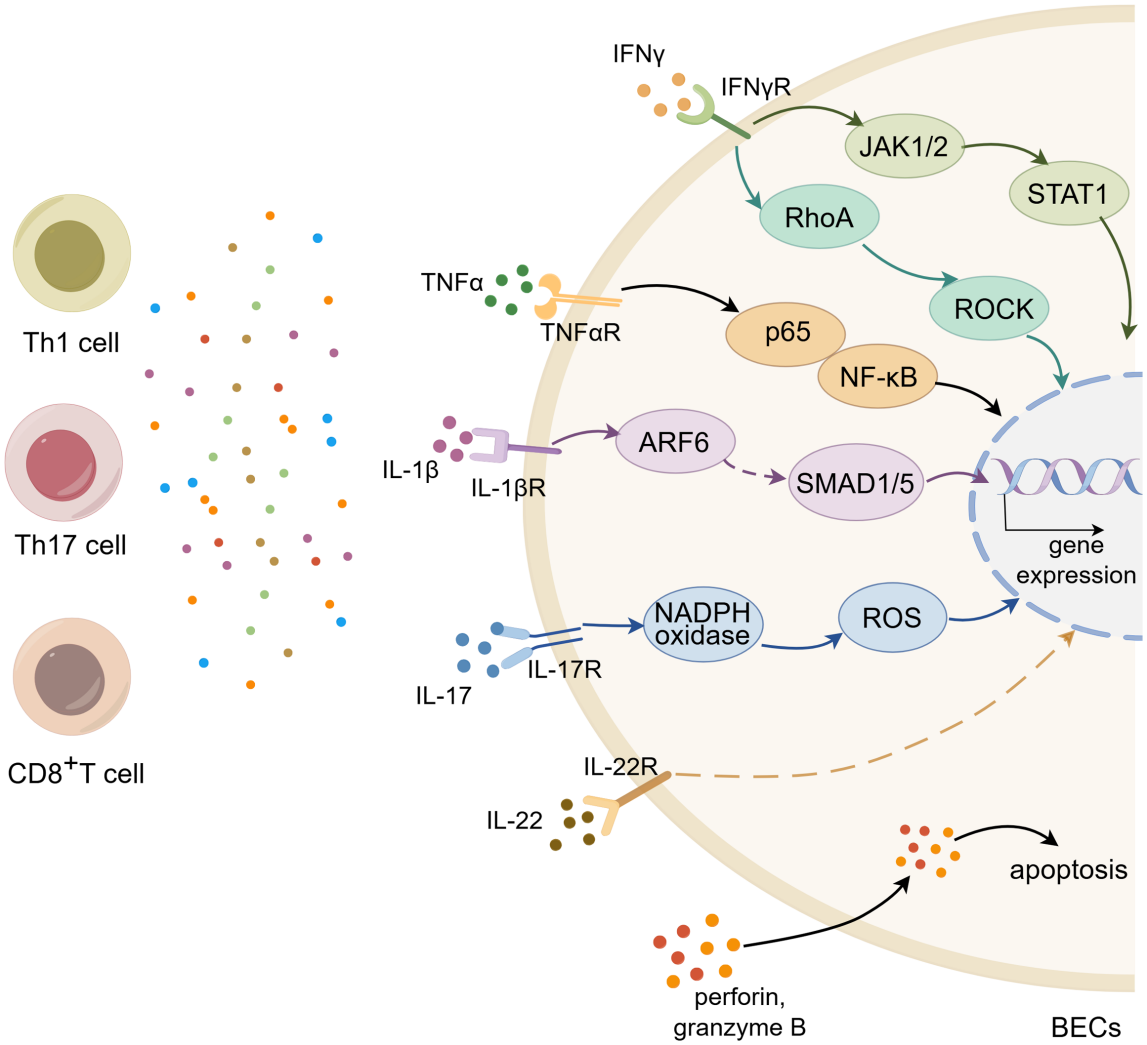
T cell impair BEC barrier function and reprogram immunomodulatory function. (Created with Home for Researchers, https://www.home-for-researchers.com/).

Prior research has illuminated the importance of myelin-specific T cells in the pathogenesis of MS and EAE. Dysfunction of BECs is considered an early hallmark of MS and serves as a pivotal factor in disease progression (52). Myelin-specific T cells secrete various cytokines, chemokines, and cytotoxic molecules that disrupt the integrity of BECs, leading to BBB breakdown and facilitating the infiltration of inflammatory cells into the CNS (Table 2; Figure 2).

**Table 2:** The regulatory effect and mechanism of T cells on BECs.

Effect	T cell-associated molecules	BEC cell-associated molecules	Ref.
Tight junctions destruction	IFN-γ IL-17, IL-22 TNF-α IL-1β IL-4	STAT1, ROCK ROS NF-κB ARF6/ALK/SMAD1/5 /	(54–56) (57, 58) (59, 60) (63) (64)
BEC apoptosis	IL-1α / /	/ / HLA	(66) (67) (69)
Immunomodulation	/ IL-17, IL-22, TNF-α / LFA-1	MHC II CCL2, CXCL1 ICAM-1, CXCL8, CXCL10 ICAM-1/ NF-κB/P-gp	(48) (57, 70) (71) (21)

### 4.1 Disruption of T cells on BECs barrier function

BEC barrier function is the structural basis of BBB. TJs and AJs are consisdered as vital protein molecules that maintain the barrier function of BECs, the abnormal expression of which leads to disturbances in BECs barrier function and BBB damage. In MS/EAE, pathogenic T cells affect the expression of TJs and AJs through the secretion of cytokines, which then impair BEC barrier function. IFN-*γ*, secreted by Th1 cells, is elevated in the blood, cerebrospinal fluid and CNS lesions of patients with MS (53). *In vivo* and *in vitro* experiments, IFN-γ was found to downregulate the expression of ZO-1, claudin5 and VE-cadherin through the STAT1 pathway, thus leading to BBB leakage (54). It was reported that Rho-associated coiled-coil containing protein kinase (ROCK) is involved in the regulation of cytoskeletal reorganization and endothelial permeability (55). Bonney et al. discovered that IFN-*γ* influences the BECs barrier properties by modulating ROCK-mediated cytoskeletal contractile (56). Kebir et al. showed that human Th17 cells preferentially promote BBB disruption compared with human Th1 cells and the strongly expression of *IL-17R* and *IL-22R* on BECs within MS lesions. BECs-derived IL-17 and IL-22 can decrease the expression of occludin and ZO-1 and disrupt intercellular tight junctions, which might be associated with endothelium contractility mediated by ROS pathway (57, 58). *In vitro* studies, TNF-*α* was found to upregulate the expression of VCAM1 and downregulate the expression of occludin and claudin5 through the NF-κB pathway, facilitating leukocyte adhesion and endothelial barrier breakdown (59, 60). Suidan et al. investigated the effect of antigen-specific CD8^+^T cells and found that CNS infiltrating antigen-specific CD8^+^T cells destroy BECs tight junctions in a non-apoptotic manner (61). It is reported that BEC barrier dysfunction and elevated vascular permeability are given rise by endothelial-to-mesenchymal transition (EndoMT) (62). Sun et al. showed that the pro-inflammatory cytokine IL-1β can induce the ARF6-ALK-SMAD1/5 pathway-mediated EndoMT, thereby promoting BBB damage and disease progression in EAE mice (63). Usually, Th2 cells and its signature cytokine IL-4 play a protective role in MS/EAE. Surprisingly, Symth et al. showed that IL-4 alters BEC morphology and destroys the integrity (64).

BEC dysfunction can also be caused by endothelial cell apoptosis, which in turn can serve important roles in MS pathogenesis (65). Tsukada et al. revealed that cytotoxic T cells derived from MS patients exert cytotoxic effects on BECs and increase the BBB permeability (66). In parallel, another study demonstrated that activated T cells can also directly kill BECs and disrupt BBB integrity. Furthermore, BEC death is associated with the number of T cells (67). Wei et al. found that lipopolysaccharide (LPS) induces the apoptosis of BECs by activating caspase-4/11-GSDMD pathway and increases cell permeability, which ultimately leads to BBB disruption (68). BECs express human leukocyte antigen (HLA) class, which can be recognized by cytotoxic T cells. In a recent study, cytotoxic CD4^+^T cells have proven function in systemic sclerosis, whereby cytotoxic CD4^+^T cells mediate endothelial cell apoptosis and drive dysfunction in a HLA-dependent manner (69).

In summary, T cells disrupt the BEC barrier through multiple pathways, including cytokine-driven downregulation of junctional proteins, induction of EndoMT, and direct cytotoxic effects leading to apoptosis. This concerted assault severely compromises BBB integrity, facilitating uncontrolled immune cell trafficking into the CNS and amplifying the inflammatory cascade in MS.

### 4.2 Influence of BEC immunomodulatory function

Beyond disrupting physical barrier function, T cells actively reshape the immunoregulatory phenotype of BECs, creating a feed-forward loop of neuroinflammation. A primary mechanism is the upregulation of antigen presentation machinery. Wheway et al. found that PBMC elevate the expression level of *MHC* class II molecules by establishing the co-culture system of PBMC and BECs (48). *In vitro* experiments demonstrated that IL-17 and IL-22 are able to promote T cell recruitment and sequent transendothelial migration, which may be associated with CCL2 secretion of BECs (57). IL-17 and TNF-*α* stimulation of BECs can promote the production of CCL2 and CXCL1, thereby facilitating the trans-endothelial migration of Th17 cells (70). Supernatants from Th1 cells can induce the expression of ICAM-1, CXCL10, and CXCL8 in BECs (71). The loss of P-gp function in blood vessels may disrupt brain homeostasis. Previous studies have shown that P-gp regulates the secretion of CCL2 to promote the migration of CD8^+^T cells, thereby exerting an immunomodulatory effect (22). Recently, Kooij et al. further explored the mechanisms regulating P-gp expression and found that activated CD4^+^T cells interaction through ICAM-1 activate the NF-κB signaling pathway, which in turn leads to P-gp dysfunction in BECs (21).

In summary, T cells do not merely passively breach the BBB but actively reprogram BECs into a pro-inflammatory state. This reprogramming includes enhanced antigen presentation, altered chemokine secretion to recruit more immune cells, and the suppression of key homeostatic functions like P-gp activity. This transformed BEC phenotype significantly contributes to the chronicity and self-sustaining nature of the neuroinflammatory process in MS.

## 5 Therapeutic strategies targeting BECs or T cells

In MS, the sustained crosstalk between BECs and T cells is critical for immune cell migration into CNS parenchyma. The immunomodulatory function of BECs facilitates T cell activation and subsequent MS progression. In turn, activated T cells further enhance the immunomodulatory capacity of BECs while impairing their barrier function, leading to worsening MS symptoms. Therefore, therapeutic strategies targeting BECs or T cells can be taken to disrupt the BECs-T cells interaction, thus effectively alleviating MS pathology.

### 5.1 Therapies targeting BECs

Given the importance of adhesion molecules in BECs-T cells interaction, developing drugs that target adhesion molecules are imperative. Glatiramer acetate is a copolymer peptide drug for the treatment of MS. It downregulates the expression of VCAM-1 and E-selectin against TNF-*α*-induced monocyte adhesion to BECs by suppressing the nuclear translocation and activation of the NF-κB pathway (72). The activation of type *Ι* IFN signaling pathway in BECs increases vascular permeability, resulting in MS progression (73). In addition to immunoregulatory effects, IFN-*β* has been shown to decrease the expression of adhesion molecules in BECs, reduce the transmigration of immune cells and protect BBB integrity *in vivo* (74, 75). Poly ADP-ribose polymerase (PARP), a member of the NAD-dependent histone deacetylase family, regulates the inflammation-associated genes. PARP inhibitors can downregulate adhesion molecule expression and upregulate TJ protein expression by inhibiting RhoA/RAC1 activity, thereby reducing leukocyte adhesion and migration across BECs (76).

Protecting BEC TJs is a critical strategy for reducing T cell transendothelial migration and preventing MS progression. It has been reported that BECs express glucocorticoid (GC) receptor. GC effectively attenuate BBB damage of MS patients through activation glucocorticoid receptor (77). Blecharz et al. showed that serum derived from MS patients downregulate the expression of occludin and claudin-5 while upregulating matrix metalloproteinase-9 (MMP-9), and these effects can be reversed by dexamethasone (78). Similarly, Forster et al. found that hydrocortisone protects against TNF-*α*-induced BECs barrier damage by upregulating the expression of occludin and claudin5 through activation of GC receptors (59). Rho/ROCK pathway is involved in the regulation of TJs. Fasudil (HA 1077), a ROCK inhibitor, increases occludin and ZO-1 expression and decreases BBB permeability, thereby preventing CNS from invasive peripheral immune cells (79).

In summary, therapeutic strategies targeting BECs primarily focus on two core objectives: first, interfering with the recognition and anchoring of immune cells by inhibiting adhesion molecules; and second, directly reinforcing the physical barrier of the BBB by enhancing the expression and function of tight junction proteins. These approaches work in concert to sever the vicious cycle of neuroinflammation at the ‘gateway’ to the CNS.

### 5.2 Therapies targeting T cells

Suppressing the proliferation and activation of pathogenic T cells is an important approach for treating MS. The signaling induced by IL-2 binding to its receptor, IL-2 receptor, on the surface of T cells is pivotal for T cell proliferation and activation. Preclinical investigations indicate that Daclizumab, a humanized monoclonal antibody, can inactivates T cells and inhibits brain inflammation in MS patients through its interaction with the *α*-chain of the IL-2 receptor (CD25) (80, 81). CD52 is widely expressed on the surface of T cells. Alemtuzumab is another humanized monoclonal therapeutic antibody that effectively targets the depletion of MS patients’ T cells, and it can reduce the mean relapse (82, 83). The *de novo* synthesis of pyrimidine nucleotides is critical for immune cell proliferation and activation. Teriflunomide can block pyrimidine synthesis through selectively suppressing dihydroorotate dehydrogenase (84). The use of Teriflunomide in MS patients can reduce the proliferation and activation of T cells (85).

Reducing the migration of peripheral T cells to the CNS can effectively control MS progression. There are three major ways to inhibit T cell migration: (1) inhibiting the migration of T cells from the lymphoid organs and (2) suppressing the rolling and adhesion of T cells on the surface of BECs and (3) exhausting the peripheral circulation T cells. It has been reported that the sphingosine 1-phosphate receptor (S1PR) is expressed on T cells and has essential roles in T cell migration out of lymph nodes. Fingolimod, acting as an S1PR modulator, causes T cells to be retained in lymph nodes, thus reducing their migration from the peripheral circulation to the CNS (86, 87). Natalizumab, a monoclonal antibody against VLA-4, is used for treating relapsing–remitting multiple sclerosis (RRMS). It can inhibit the rolling and adhesion of T cells on the surface of BECs and reduce the infiltration of T cells into CNS by blocking VLA-4/VCAM1 interactions (85). An other study found that The transmembrane protein DICAM preferentially promotes the transendothelial migration of Th17 cells. Monoclonal antibodies targeting DICAM can selectively block the adhesion and extravasation of Th17 cells, thereby effectively alleviating neurological deficits in EAE (38). Essen et al. demonstrated that CD20^+^T cells exhibit a high proliferative capacity in response to CNS antigens, and the number of CD20^+^T cells in CSF is correlated with MS disease severity (88). Ocrelizumab deplete CD20^+^T cells via targeting CD20 (89).

In summary, therapies targeting T cells intervene at multiple stages of their pathogenic process: suppressing their activation and proliferation at the source, preventing their migration and infiltration into the CNS, or directly depleting pathogenic T cell subsets in the circulation. The common goal of these strategies is to reduce the accumulation of aggressive T cells within the CNS, thereby controlling inflammation and alleviating the disease.

## 6 Discussion

**Figure 3:**
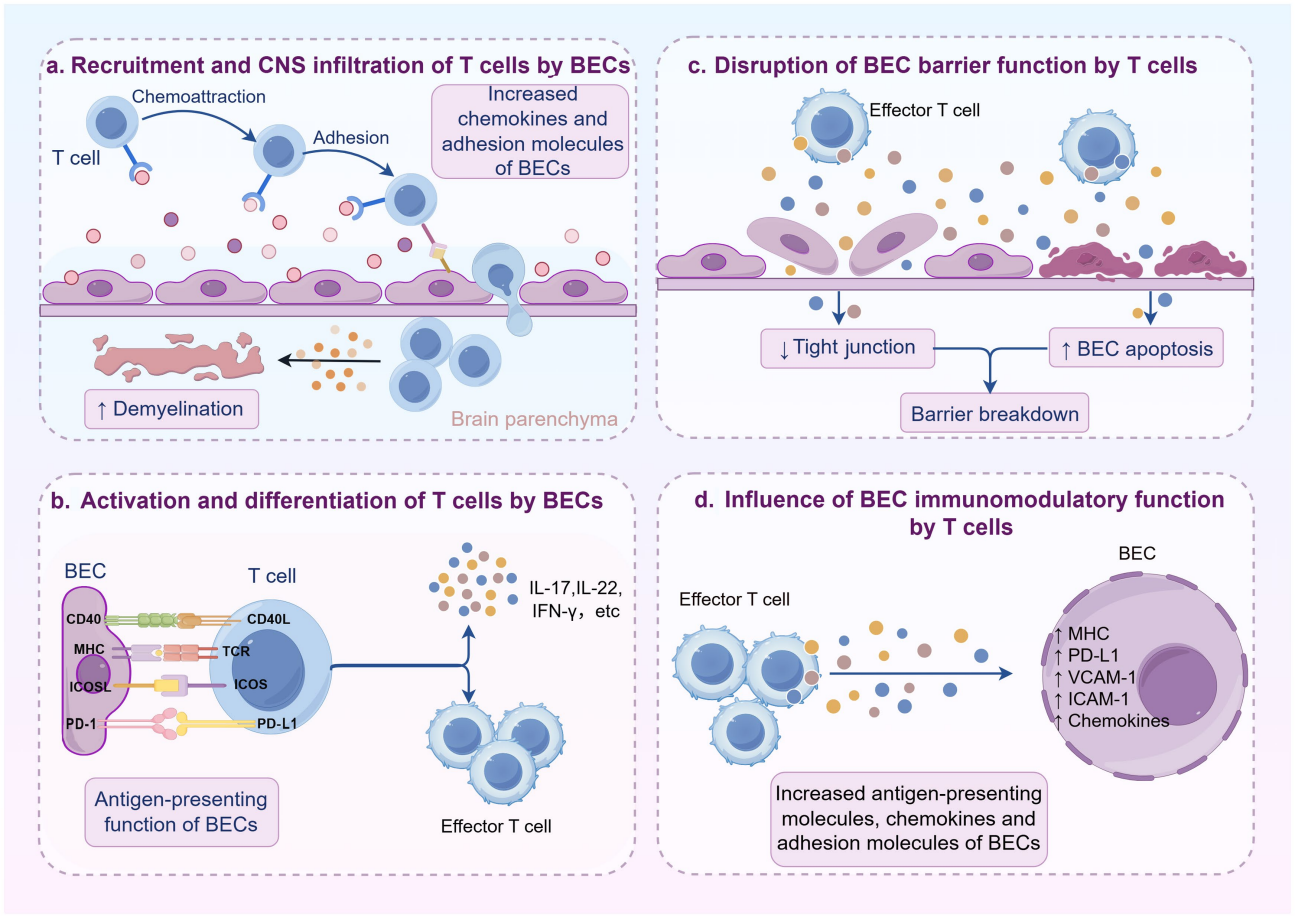
Mechanism diagram of interaction between BECs and T cells. **(a)** Under inflammatory conditions, BECs are activated and upregulated the expression of chemokines and adhesion molecules, which mediates the recruitment and CNS infiltration of T cells. **(b)** Activated BECs express high levels of MHC molecules and costimulatory molecules, facilitating T cell activation and differentiation. **(c)** Activated T cells disrupt tight junction between BECs and induce apoptosis by secreting proinflammatory cytokines and cytotoxic factors, ultimately leading to increased BBB permeability and functional impairment. **(d)** Activated T cells enhance the immunoregulatory function of BECs by secreting cytokines that regulate the upregulation of antigen-presentation-related molecules and T cell recruitment-related molecules on BECs (Created with Home for Researchers, https://www.home-for-researchers.com/).

In this review, we primarily examined the interaction between BECs and T cells, revealing that their reciprocal relationship fuels the development and progression of MS (Figure 3). This interaction is pivotal, as it enables the diapedesis of peripheral immune cells into the CNS, ultimately exacerbating neuroinflammation. Consequently, exploring therapeutic strategies aimed at BECs or T cells offers promise in improving MS’s pathological course by disrupting the intricate interaction between these two cell types. This review summarizes the current clinical therapies aimed at BECs or T cells, emphasizing their ability to disrupt the interaction between these cells. However, there are limitations in existing research that hint at opportunities for future advancements.

First, although therapeutics strategies to suppress T cell infiltration (such as natalizumab) have shown significant efficacy in controlling MS disease activity, the associated risk of severe opportunistic infections like progressive multifocal leukoencephalopathy (PML) underscores the fundamental limitations of non-selective immunosuppression. This risk primarily stems from a comprehensive weakening of CNS immune surveillance, particularly affecting immune cell subsets essential for combating pathogens such as the JC virus. Future treatment strategies must shift toward greater precision and personalization. Accumulating evidence indicates significant heterogeneity in pathogenic T cells among MS patients and across different disease stages. For instance, molecules such as DICAM and integrin α3 have been found to be specifically expressed on the surface of pathogenic Th17 cells and are involved in their CNS infiltration process; selectively blocking these molecules can effectively suppress their pathogenicity while avoiding broad immunosuppression. Future research directions should focus on: first, developing next-generation biologics capable of specifically interfering with the function or CNS homing of specific pathogenic subsets (such as targeting Th17-related cytokine receptors or their specific surface markers); second, distinguishing between Th1-dominant and Th17-dominant patients based on blood or cerebrospinal fluid biomarkers to guide clinical medication. This pathology biology-based personalized treatment strategy holds promise for precisely controlling neuroinflammation while maximally preserving beneficial immune surveillance functions, thereby reducing the side effects of systemic immunosuppression.

Secondly, while existing studies have uncovered some molecular mechanisms underlying BEC dysfunction, such as EndoMT and tight junction disruption, these findings are insufficient to fully elucidate the pathological mechanisms. This knowledge gap primarily stems from the difficulty of conducting direct *in vivo* studies on the CNS. Current research relies on animal models, which often fail to fully recapitulate the complex pathophysiological characteristics of the human neurovascular unit. To overcome this challenge, researchers are developing co-culture models using induced pluripotent stem cell-derived BECs alongside pericytes and astrocytes, which can partially replicate the structure and function of the neurovascular unit. It is also important to note that pericytes, as integral components of the BBB, directly participate in T cell CNS infiltration through the expression of adhesion molecules. Furthermore, pericytes express MHC molecules and can regulate T cell proliferation and activation by presenting antigens. Building on this foundation, forthcoming research should employ advanced techniques, including single-cell sequencing, gene editing, and high-throughput screening, to pinpoint crucial molecular pathways and regulatory mechanisms linked to human BECs and pericytes. This approach will enhance our comprehension of blood–brain barrier breakdown and establish fundamental theories and targets for the development of innovative BEC-modulating drugs.

It is noteworthy that there is currently a lack of reliable clinical biomarkers to quantify the severity of cerebrovascular endothelial cell and T-cell interactions in patients. Although pathological analysis of postmortem brain tissue remains the gold standard for assessment, it is inherently unsuitable for longitudinal monitoring or clinical decision-making. Therefore, developing non-invasive biomarkers is crucial. Promising research directions include: profiling the spectra of cell adhesion molecules (such as soluble ICAM-1 and VCAM-1) or other endothelial cell-derived factors in cerebrospinal fluid (CSF) or peripheral blood, as these indicators may correlate with cerebrovascular inflammation. Additionally, advanced neuroimaging techniques can enable real-time assessment of blood–brain barrier integrity. The successful identification and validation of such biomarkers would revolutionize patient stratification and management, enable early detection of neuroinflammatory events, and provide much-needed pharmacodynamic endpoint measures for clinical trials targeting the neurovascular unit.

In summary, the interplay between BECs and T cells is a core component of MS pathology and warrants further investigation. Future research should focus on the distinct interactions between specific T-cell subtypes (such as Th1, Th17, and CD8^+^ T cells) and BECs. This precise, subset-based understanding will provide critical theoretical guidance for developing new therapeutics that specifically target pathogenic cells while preserving protective immune functions, ultimately leading to more effective and safer novel treatment strategies for MS patients.
